# *In vitro* reduction of enamel erosion by sugarcane-derived cystatin associated with sodium trimetaphosphate

**DOI:** 10.1590/1807-3107bor-2024.vol38.0124

**Published:** 2024-12-09

**Authors:** Carolina Ruis Ferrari, Karolyne Sayuri de Araujo Kitamoto, Vinicius Taioqui Pelá, Éven Akemi Taira, Tamara Teodoro Araújo, Larissa Tercilia Grizzo Thomassian, Flávio Henrique-Silva, Juliano Pelim Pessan, Marília Afonso Rabelo Buzalaf

**Affiliations:** (a) Universidade de São Paulo – USP, Bauru School of Dentistry, Department of Biological Sciences, Bauru, SP, Brazil.; (b) Universidade Federal de São Carlos – UFSCar, Department of Genetics and Evolution, São Carlos, SP, Brazil.; (c) Universidade Estadual Paulista – Unesp, School of Dentistry, Department of Preventive and Restorative Dentistry, Araçatuba, SP, Brazil.

**Keywords:** Cystatins, Dental Pellicle, Saliva, Tooth Erosion

## Abstract

The objective of this *in vitro* study was to assess the efficacy of CaneCPI-5, either alone or in combination with various concentrations of sodium trimetaphosphate (TMP) in protecting against initial enamel erosion. A total of 135 bovine enamel specimens were prepared and categorized into nine groups (n/group=15) according to the following treatments: Deionized water; Commercial solution (Elmex Erosion Protection^TM^); 0.1 mg/mL CaneCPI-5; 0.5% TMP; 1.0% TMP; 3.0% TMP; 0.1 mg/mL CaneCPI-5+0.5% TMP; 0.1 mg/mL CaneCPI-5+1.0%TMP; and 0.1 mg/mL CaneCPI-5+3.0%TMP. The specimens were treated with the respective solutions for 2 h, followed by acquired enamel pellicle formation for 2 h and exposure to 0.65% citric acid (CA) for 1 min. These procedures were repeated once a day for three consecutive days. Demineralization was assessed by the percentage change in surface hardness (%CSH) and calcium release into CA, analyzed by the Arsenazo III method. The data were evaluated using Kruskal-Wallis/Dunn's tests. Regarding %CSH, CaneCPI-5+3.0%TMP was the most effective treatment when compared to the CaneCPI-5 group alone. As for calcium release into CA, the CaneCPI-5+0.5% TMP and CaneCPI-5 groups (both with lower calcium release) did not significantly differ from the commercial solution. In conclusion, combination of CaneCPI-5 with TMP enhances the protective potential against initial enamel erosion *in vitro.*

## Introduction

Erosive tooth wear (ETW) consists of the progressive loss of mineralized tooth substance with dental erosion as the primary causal factor.^1,2^ The acids involved in this process are of non-bacterial origin^2^ and can be either intrinsic^3^ or extrinsic.^4^ In the earliest stages of dental erosion, only surface softening occurs.^5^ With increased acid exposure, loss of volume and fine subsurface softening take place.^5^ ETW has a significant prevalence in the population,^6^ and there is a consensus that the severity of ETW increases with age.^7^ An individual's key protective factor against ETW is saliva, mainly because of its role in acquired enamel pellicle (AEP) formation.^8^

AEP is a bacteria-free film composed of salivary proteins, glycoproteins, and lipids that are adsorbed onto the dental surface, forming a highly organized and dynamic film.^9^ This protective layer functions as a mechanical barrier against acids on the dental surface, reducing demineralization.^10^ It is known that the pellicle's protective effectiveness is determined by its composition and physical properties, such as thickness.^11^ Importantly, proteins in the basal layer of the AEP remain intact, even after prolonged erosive challenges.^12^ Drawing on this concept, a new protection strategy against ETW was developed, based on "acquired pellicle engineering," which consists in reinforcing the basal layer of the AEP with acid-resistant proteins.^13,14^

Among these acid-resistant proteins, cystatin-B has been identified^15^ as a promising option against ETW. Nonetheless, because of the high cost of the human recombinant protein, it is considered commercially impractical. Therefore, our group identified and characterized a new sugarcane-derived cystatin (CaneCPI-5) that has a strong binding affinity for enamel.^16^ This protein demonstrated a reduction in enamel surface free energy, with a more negative polar component,^17^ which may influence AEP formation, owing to the change in protein composition. *In vitro*,^16^ *in situ*,^18^ and *in vivo* ^13,19^ studies have demonstrated the high potential of this recombinant protein to protect dental enamel from ETW-causing acids.

The addition of sodium trimetaphosphate (TMP) to fluoride vehicles promotes a significant increase in their ability to favorably interfere in demineralization and remineralization of enamel induced by cariogenic and erosive challenges, even when associated with low-concentration fluoride varnishes.^20,21^ Such effects are attributed to its high adsorption capacity to enamel, consequently reducing its solubility, in addition to having a strong tendency to form complexes with cations, due to the presence of a phosphate group in the molecular structure of TMP.^22,23^ The action of TMP is associated with: a) significant reduction in enamel mineral loss both *in vitro* and *in vivo*; b) reduced formation of extracellular polysaccharides in the dental biofilm formed *in situ*; c) increase in fluoride, calcium, and phosphate concentrations in enamel and dental biofilm; d) increase in the remineralization rate of previously demineralized dental enamel; and e) dental erosion reduction.^24–27^ This is associated with a decrease in the enamel surface free energy and its nonpolar component, coupled with an increase in the number of electron donor sites, which promotes greater adsorption of Ca^2+^ and PO_4_^3-^ ions.^24^ Despite the lack of data on the effects of TMP on the AEP, evidence indicates that the effects of this phosphate on enamel are related to changes in AEP composition, as a result of its high capacity of adsorption to enamel^22,23^ and alteration of the surface free energy of this substrate.^28^

Thus, the association of CaneCPI-5 (organic component) with an inorganic component such as TMP may be an alternative to enhance the effect of protein against ETW, given the synergistic effect observed when CaneCPI-5 is combined with sodium fluoride (NaF).^29^ Therefore, the aim of this study was to evaluate the effect of CaneCPI-5 associated or not with different concentrations of TMP on the protection against initial enamel erosion *in vitro*. The null hypothesis to be tested is that CaneCPI-5 associated or not with TMP would not have a protective effect against initial enamel erosion *in vitro*.

## Methods

### Ethical considerations

The protocol of this study was approved by the Institutional Human Research Ethics Committee (protocol No. 64568622.2.0000.5417) and by the Ethics Committee on the Use of Animals (protocol 009/2022)

### CaneCPI-5 production

CaneCPI-5 was synthesized using the recombinant bacterial strain *Escherichia coli* Rosetta (DE3), as previously described.^16,30^ Briefly, the bacterial strain was transformed with the plasmid pET28aCaneCPI-5. The expressed protein was purified from the soluble fraction of bacterial cultures induced by IPTG (Isopropyl-β—Dt-hiogalactoside), centrifuged, and sonicated. Purification was performed by affinity chromatography using columns containing Ni-NTA Superflow nickel resin (Qiagen).

### Specimen preparation

The study by Santiago et al.^16^ was used for sample size calculation. A minimum detectable difference in %CSH of 27% was considered, with a standard deviation of 9%, a type α error of 5%, and a type β error of 20%. Therefore, 15 specimens were included.

One hundred and thirty five bovine enamel specimens (4 x 4 x 3 mm) were prepared from the buccal surface of bovine incisors. The crowns of the bovine teeth were individually mounted on acrylic plates (40 x 40 x 5 mm^3^) screwed into an ISOMET Low-Speed Saw precision cutting device (Buehler, Lake Bluff, USA). The specimens were cut with two double-sided diamond discs: XL 12205, "High concentration," 102 x 0.3 x 12.7 mm^3^ (Extec Corp., Ref: 12205, Enfield, USA) and a stainless-steel spacer (diameter: 7 cm, thickness: 4 mm, and central hole: 1.3 cm) between the discs.

After the samples were cut, they were polished. To achieve this, they were fixed with sticky Kota wax using a PKT instrument (Kota Ind., São Paulo, Brazil) and a lamp (JON, Ind. Bras., Juiz de Fora, Brazil) at the center of an acrylic disc (30 mm in diameter and 8 mm in thickness), with the enamel surface facing the disc, with the intention of initially planing the internal dentin. The assembly (disc/tooth) was mounted on a metallographic polisher (Arotec, São Paulo, Brazil) for dentin planing, allowing parallelism between the polished surfaces and the acrylic base to which the specimens were attached. A 320-grit silicon carbide sandpaper was then used under cooling with deionized water until the specimens reached a thickness of approximately 2.5 to 3 mm. The polisher was operated at a low speed for 30 s to 3 min until the desired thickness was achieved. Subsequently, the specimens were removed from the acrylic disc and the discs were cleaned with xylene (Merck, Darmstadt, Germany) to remove any residual wax adhered to them. Thereafter, the enamel specimens were fixed again with sticky wax at the center of the acrylic plate with the enamel surface exposed, for polishing, thus obtaining a surface that was flat and parallel to the base, essential for the tests. Again, the assembly was mounted on the polisher and the surface was initially worn down with a 600-grit silicon carbide sandpaper under cooling with deionized water for approximately 2 min (removal of approximately 150–200 μm of tissue), using two weights, at low speed. Polishing was performed with a 1200-grit silicon carbide sandpaper (Extec Corp., Enfield, USA) under cooling for 2-4 min, applying two weights, at high speed. This was followed by polishing with felt and diamond solution for 2 – 4 min, also applying two weights. The wear on the enamel surface was controlled using a micrometer to prevent excessive wear that could reach the dentin (region of non-interest in the present study). All specimens underwent this wear control, with measurements taken before and after polishing, and the difference between the initial and final data was employed to calculate the total wear value. In addition, some representative enamel specimens were examined under the microscope to confirm the absence of dentinal tubules, indicating that the specimens were within the dentin (region of non-interest in the present study). Thus, specimens that exceeded the parameters of enamel tissue removal and those in which the wear reached the dentin region were discarded. The specimens were cleaned in distilled water in an ultrasonic bath and finally stored at 4°C until the start of the experiments.

### Human saliva collection

Nine volunteers (five men and four women) aged 20 to 38 years were selected. The following inclusion criteria were adopted: nonsmokers, individuals with no history of long-term medication use, nonpregnant women, individuals with no systemic diseases and with good oral health, individuals without active caries, individuals without periodontal disease, and those with normal salivary flow rates (stimulated saliva > 1.0 mL/min and unstimulated saliva > 0.3 mL/min). Volunteers provided stimulated saliva after chewing paraffin wax for 10 min, from 9:00 a.m. to 10:00 a.m, with samples stored on ice afterwards. Collected saliva was pooled and centrifuged for 20 min at 14,000 g and stored at 4°C.^31^ The supernatants were collected and stored at −80°C until the start of the experiment.

### Treatment, incubation in human saliva, and acid challenge

Bovine enamel specimens were randomly distributed into nine groups (n = 15/group) according to the treatments: a) Deionized water (negative control); b) Commercial solution containing SnCl_2_/NaF/AmF (800 ppm Sn^+2^ + 500 ppm F^-^, pH 4.5, Elmex Erosion Protection® – GABA International AG (positive control 1); c) 0.1 mg/mL CaneCPI-5 (positive control 2); d) 0.5% TMP; e) 1.0% TMP; f) 3.0% TMP; g) 0.1 mg/mL CaneCPI-5 + 0.5% TMP; h) 0.1 mg/mL CaneCPI-5 + 1.0% TMP; and i) 0.1 mg/mL CaneCPI-5 + 3.0% TMP. All treatments, except for the commercial solution, had the same consistency, color, and smell.

To ensure standardization of the exposed area, bovine enamel blocks were placed in 96-well plates with the enamel surface facing upwards, making sure that the entire enamel surface was immersed in the treatment solution. To prevent exposure of the lateral and bottom walls, these surfaces were protected with red nail varnish (Risqué®, São Paulo, Brazil).

**Figure 1:**
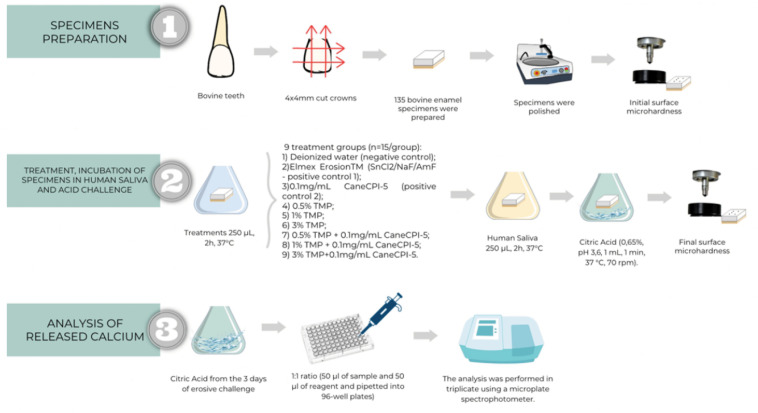
*In vitro* experimental protocol and calcium analysis.

Initially, the samples were individually treated with the respective solutions (250 μL, 2 h, 37°C, under constant agitation). The acquired pellicle was then individually formed by adding human saliva to each specimen (250 μL, 2 h, 37°C, under constant stirring). Subsequently, the specimens were individually subjected to the erosive challenge by immersion in 0.65% citric acid (pH 3.6, 1 mL, 1 min, 37°C, 70 rpm). Between each application (treatment, AEP formation, and acid challenge), the specimens were carefully washed with deionized water (10 s) and dried (5 s). These procedures were performed once a day for three consecutive days to simulate initial erosion.^32^ During brief interims and overnight, the specimens were stored in humidity chambers (4°C) (Figure 1).

### Percentage change in surface hardness

Measurements were performed using a Knoop diamond indenter with a load of 50 g and a dwell time of 15 s (HMV-2, Buehler, Lake Bluff, USA). Five indentations per specimen were made (spaced 150 μm apart on a defect-free surface) at the beginning of the experiment (SH_initial_) and after the final erosive challenge (SH_final_). The percentage change in surface hardness (%CSH) was calculated as a measure of enamel softening, according to the following equation: %CSH = [(SH_initial_-SH_final_)/SH_initial_]x100 for statistical analyses.

### Analysis of released calcium

The amount of calcium present in the citric acid after the erosive challenges was individually analyzed by the Arsenazo III colorimetric method (Sigma Aldrich, CAS 1668-00-4, Burlington, USA,).^33^ The reagent was diluted with part of the sample or calcium standard, in a 1:1 ratio (50 μL of sample and 50 μL of reagent) and pipetted into 96-well plates. A calibration curve was constructed so that the absorbances remained within the linearity range of 0-1 using different concentrations of calcium. Absorbances were analyzed at 650 nm, at 25°C. The analyses were performed in triplicate on a microplate spectrophotometer (Biotek Instrumentals, Inc., Winooski, USA) (Figure 1).

### Statistical analysis

GraphPad Prism software (version 6.0 for Windows; GraphPad Software Inc., La Jolla, USA) was used. Data were checked for normality (Kolmogorov-Smirnov test) and homogeneity (Bartlett test) and were analyzed by the Kruskal-Wallis test, followed by Dunn's test. Results are presented as median and interquartile range. The significance level was set at 5% (two-tailed).

## Results

### Change in surface hardness

After three consecutive days CaneCPI-5 and all combinations of Cane-CPI-5 + TMP significantly protected the enamel when compared to the negative control and did not show significant difference when compared to Elmex Erosion^TM^. The isolated TMP groups did not provide a protective effect when compared to deionized water.

**Figure 2:**
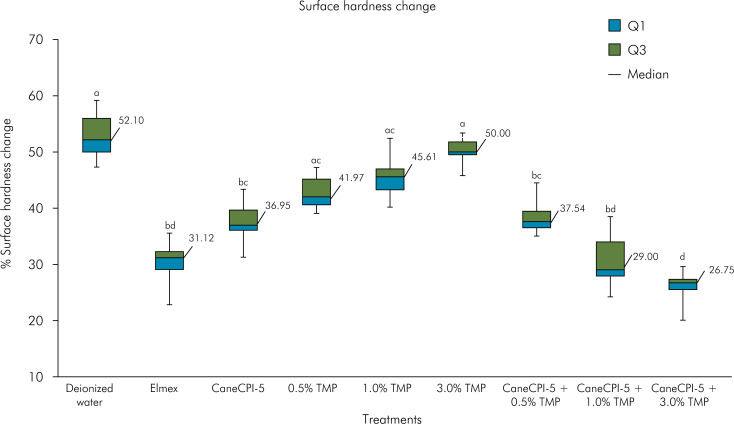
Percentage change in surface hardness after treatments of bovine enamel specimens with 0.1 mg/mL CaneCPI-5 alone, TMP alone at different concentrations or their combination, followed by acquired enamel pellicle formation for 2 h and erosive challenges with 0.65% citric acid at pH 3.6 for 1 min.

CaneCPI-5 + 3% TMP (26.75%; 1.82%) was the most effective treatment. It did not show significant differences when compared with Elmex (31.12%; 3.19%) or CaneCPI-5 + 1.0% TMP (29.00%; 6.20%), but performed significantly better than all the other groups, including CaneCPI-5 and TMP alone, regardless of the concentration (p < 0.05) (Figure 2). Table 1 shows the confidence interval values (95%CI).

Treatments were performed daily for 3 days to simulate initial erosion. Different letters show significant differences among the treatments (Kruskal-Wallis and Dunn's tests, p < 0.05) [median (interquartile-II range)], n = 15. *Q1* quartile1, *Q3* quartile 3.

**Table 1:** Effect size and confidence interval (CI) for change in *surface hardness*.

Groups	Minimum – Maximum (95%CI)
Deionized water	47.31–59.25
Commercial solution (Elmex Erosion Protection^TM^)	22.82–35.48
0.1 mg/mL CaneCPI-5	31.21–43.33
0.5% TMP	39.01–47.16
1.0% TMP	40.09–52.46
3.0% TMP	45.82–53.41
0.1 mg/mL CaneCPI-5+0.5% TMP	35.01–44.41
0.1 mg/mL CaneCPI-5+1.0% TMP	24.12–38.46
0.1 mg/mL CaneCPI-5+3.0% TMP	20.01–29.60

### Calcium analysis by the Arsenazo III colorimetric method

**Figure 3:**
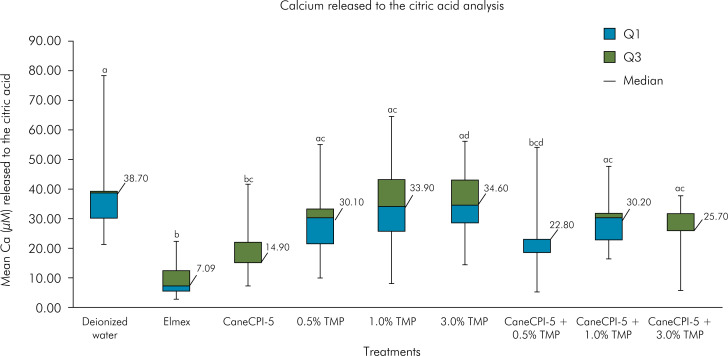
Calcium released into the citric acid (μM) by the Arsenazo III method. Bovine enamel specimens were treated with 0.1 mg/mL CaneCPI-5 alone, TMP alone at different concentrations or their combination, followed by acquired enamel pellicle formation for 2 h and erosive challenges with 0.65% citric acid at pH 3.6 for 1 min.

The groups with lowest calcium release into the citric acid were Elmex (7.09 μM; 6.97μ), CaneCPI-5 alone (14.90 μM; 7.04 μM), and CaneCPI-5 + 0.5% TMP (22.80 μM; 4.35 μM). These groups did not show significant differences among themselves, but they released significantly lower amounts of calcium when compared to the negative control (38.70 μM; 9.16 μM). All the other experimental groups did not significantly differ from the negative control (Figure 3). Table 2 shows the confidence interval values (95%CI).

Treatments were performed daily for 3 days. Different letters show significant differences among the treatments (Kruskal-Wallis and Dunn's tests, p < 0.05) [median (interquartile-II range)], n = 15. *Q1* quartile1, *Q3* quartile 3.

**Table 2:** Effect size and confidence interval (CI) for calcium analysis by the Arsenazo III colorimetric method.

Groups	Minimum – Maximum (95%CI)
Deionized water	0.0212–0.0784
Commercial solution (Elmex Erosion Protection^TM^)	0.0025–0.0221
0.1 mg/mL CaneCPI-5	0.0004–0.0415
0.5% TMP	0.0098–0.0549
1.0% TMP	0.0080–0.0646
3.0% TMP	0.0142–0.0560
0.1 mg/mL CaneCPI-5+0.5% TMP	0.0051–0.0540
0.1 mg/mL CaneCPI-5+1.0% TMP	0.0163–0.0476
0.1 mg/mL CaneCPI-5+3.0% TMP	0.0056–0.0377

## Discussion

In recent decades, extensive research has explored ways to enhance the protective effect of active agents against erosive tooth wear. TMP, in particular, has been suggested as an enhancer of the effect of fluoride, whether associated with mouthwashes,^28^ varnishes,^34^ or toothpastes.^35^ Considering the promising results of these studies, in which the addition of TMP to these fluoride-containing vehicles promoted a significant increase in the therapeutic potential of fluoride, in the present study, we proposed the addition of TMP to CaneCPI-5, a sugarcane-derived phytocystatin, which has shown good results against erosive demineralization.^16^ Our study corroborates the protective effect of the association between phosphate and protein, demonstrating that the association between CaneCPI-5 and TMP at the concentration of 3.0% yielded the best result . This association significantly decreased surface hardness loss compared to both CaneCPI-5 alone and TMP alone, regardless of the concentration. The absence of a significant effect of TMP alone was expected, as reported previously.^36^ Consequently, the null hypothesis of the study was rejected.

To interpret calcium analysis results, it is crucial to consider various factors. The AEP can release calcium into an acidic environment.^37^ This released calcium adds to the calcium released from hydroxyapatite. During treatments that involve acquired pellicle engineering, caution is advised when interpreting this response variable. This is because, during the engineering process, proteins with varying calcium-binding capabilities might replace the pellicle's initial proteins, affecting the outcomes across different groups. Notably, CaneCPI-5 + 0.5% TMP showed the most favorable outcome in calcium analysis, presenting intriguing results. Hence, the use of the Arsenazo III method in this analysis could have been influenced by the presence of calcium within the AEP. Therefore, given these considerations, the %CSH analysis might provide more reliable results.

Regarding the concentrations and particle sizes of the TMP employed in the present study, initial research shows no difference in the effectiveness of TMP associated with fluoride at the concentrations between 1.0% and 2.0%.^34^ Therefore, concentrations of 0.5%, 1.0%, and 3.0% were used in this study. Additionally, although nanoparticulate TMP shows better results and can be used at lower concentrations,^28^ in the present study, microparticulate TMP was employed, given that this is the first study to associate phosphate with protein. Furthermore, a previous study has shown that micrometric particles associated with fluoride toothpaste were more effective against dental erosion compared to groups that did not include phosphate.^38,39^ However, it should be noted that the hypothesis suggests smaller TMP particles may react more effectively with the enamel surface, potentially leading to a significant reduction of enamel erosion.^21^ Therefore, future studies should be conducted with nanometric particles.

**Figure 4:**
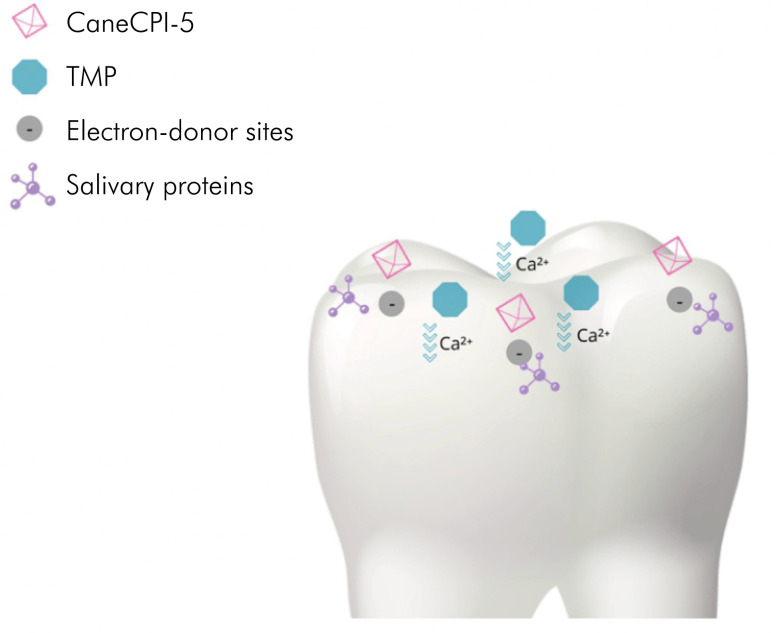
Representation of the interaction of CaneCPI-5 and TMP with the tooth surface and their effects: increase in electron donor sites that favor the adsorption of salivary proteins and increase in the diffusion of calcium ions, respectively.

Both TMP^22,23^ and CaneCPI-5^16^ have a high binding force to enamel and, once bound, they reduce the surface free energy of this substrate.^16,28^ In the case of TMP, the reduction of surface free energy facilitates the diffusion of Ca^+2^. CaneCPI-5 also increases the electron-donor sites on the enamel surface.^16^ This favors the adsorption of cationic species, such as Ca^2+^ and Ca(H_2_PO_4_) (calcium phosphate), as well as cationic acid-resistant salivary proteins, helping to explain the reduction in %CSH. The enhanced adsorption of Ca(H_2_PO_4_)_2_ by CaneCPI-5 helps to explain why the association of protein with lower concentrations of TMP was less effective than the higher concentration (3%) (Figure 4).

Although the *in vitro* protocol has been considered appropriate for preliminary investigations and for screening of new potential products,^32^ it does not accurately reflect the clinical setting. The main obstacle lies in the exposure time to protein solutions (2 h), required because of the unique combination of components. In dental practice, mouthwashes are specifically used for just 1–2 min. Furthermore, this study did not include a comprehensive assessment of erosive tooth wear because the effect of abrasion-related components of toothbrushing was not assessed. Therefore, additional studies are needed to evaluate the effect of protein solutions at time intervals that more closely reflect clinical practice. Moreover, the static model utilized in this study, in which the same saliva was in contact with the specimens for 2 h to form the AEP, contrasts with the clinical practice. In the clinical setting, saliva is continuously produced and removed, potentially impacting protein absorption and effectiveness of the protection system. Further studies evaluating the effect of abrasion and *in vivo* studies examining the impact of this combination of components in the AEP protein profile should be considered.

## Conclusion

In conclusion, our study showed that combining the organic component, CaneCPI-5, with the inorganic component, TMP, effectively reduced initial enamel erosion *in vitro*, particularly in relation to the loss of surface hardness. To further explore the potential of CaneCPI-5 and TMP in preventive dentistry, future research should employ methodologies more closely aligned with the clinical setting. This includes *in vivo* study designs, shorter treatment durations, and the incorporation of abrasives, which are vital for facilitating the practical application of this combination.
